# Elovl5 Expression in the Central Nervous System of the Adult Mouse

**DOI:** 10.3389/fnana.2021.669073

**Published:** 2021-04-29

**Authors:** Ilaria Balbo, Francesca Montarolo, Enrica Boda, Filippo Tempia, Eriola Hoxha

**Affiliations:** ^1^Neuroscience Institute Cavalieri Ottolenghi (NICO), Turin, Italy; ^2^Department of Neuroscience, University of Torino, Turin, Italy; ^3^Department of Molecular Biotechnology and Health Sciences, University of Torino, Turin, Italy; ^4^National Neuroscience Institute (Italy), Turin, Italy

**Keywords:** Elovl5, central nervous system, PUFA, spinocerebellar ataxia, glia, neurons

## Abstract

*ELOVL5* (Elongase of Very-Long Fatty Acid 5) gene encodes for an enzyme that elongates long chain fatty acids, with a marked preference for polyunsaturated molecules. In particular, it plays an essential role in the elongation of omega-3 and omega-6 fatty acids, precursors for long-chain polyunsaturated fatty acids (PUFAs). Mutations of *ELOVL5* cause the spino-cerebellar ataxia type 38 (SCA38), a rare autosomal neurological disease characterized by gait abnormality, dysarthria, dysphagia, hyposmia and peripheral neuropathy, conditions well represented by a mouse model with a targeted deletion of this gene (*Elovl5^–/–^* mice). However, the expression pattern of this enzyme in neuronal and glial cells of the central nervous system (CNS) is still uninvestigated. This work is aimed at filling this gap of knowledge by taking advantage of an Elovl5-reporter mouse line and immunofluorescence analyses on adult mouse CNS sections and glial cell primary cultures. Notably, Elovl5 appears expressed in a region- and cell type-specific manner. Abundant Elovl5-positive cells were found in the cerebellum, brainstem, and primary and accessory olfactory regions, where mitral cells show the most prominent expression. Hippocampal pyramidal cells of CA2/CA3 where also moderately labeled, while in the rest of the telencephalon Elovl5 expression was high in regions related to motor control. Analysis of primary glial cell cultures revealed Elovl5 expression in oligodendroglial cells at various maturation steps and in microglia, while astrocytes showed a heterogeneous *in vivo* expression of Elovl5. The elucidation of Elovl5 CNS distribution provides relevant information to understand the physiological functions of this enzyme and its PUFA products, whose unbalance is known to be involved in many pathological conditions.

## Introduction

Polyunsaturated fatty acids (PUFAs) are fatty acids containing two or more double bonds on their carbon chain, and are classified, depending on the position of the first double bond from the methyl terminal end, in ω-6 PUFAs and ω-3 PUFAs. In addition to a structural role in membranes, various PUFA metabolites also possess signaling functions, including roles in neurogenesis, neuronal survival, synaptic activity and regulation of brain inflammation (Guillou et al., 2010; Bazinet and Layé, 2014; Serhan et al., 2014). These multiple actions are the basis of the positive outcomes of PUFA-targeting treatments in various CNS pathological conditions, such as Parkinson’s disease, Alzheimer’s disease and Multiple sclerosis (Bousquet et al., 2011; Yanai, 2017; Penesová et al., 2018; Hernando et al., 2019). In mammalian organisms, essential fatty acids have to be introduced through the diet as precursors and then metabolized into more complex derivatives, by elongation and desaturation reactions performed by different enzymes located in the endoplasmic reticulum (ER) (Moon et al., 2009). A key and rate-controlling role is played by ELOVLs (ELOngation of Very Long-chain fatty acids), which are elongase enzymes responsible for and initial condensation reaction necessary to elongate fatty acids (Leonard et al., 2004; Jakobsson et al., 2006; Sassa and Kihara, 2014).

Seven ELOVL multi-pass transmembrane proteins have been identified (ELOVL1-7) and each of them shows a particular substrate preference: ELOVL1, ELOVL3, ELOVL6, and ELOVL7 elongate saturated or monounsaturated fatty acids, while ELOVL2, ELOVL4, and ELOVL5 are more selective for very long chain fatty acids (VLCF) and PUFAs (Moon et al., 2009). ELOVL5, in particular, has the unique property to condense a wide selection of PUFAs from 18 to 20 carbon atoms, including linoleic (C18:2, n-6) acid to produce arachidonic acid (C20:4, n- 6), α-linolenic (C18:3, n-3), and stearidonic (C18:4, n-3) to produce eicosapentaenoic (EPA, C20:5, n-3) and docosahexaenoic (DHA, C22:6, n-3) acids (Leonard et al., 2000; Moon et al., 2009; Shikama et al., 2015).

Mutations in ELOVL genes has been linked to various nervous system diseases. Indeed, ELOVL1 mutations lead to hypomyelination, neuro-ichthyosis, spastic paraplegia and optic atrophy (Kutkowska-Kaźmierczak et al., 2018; Mueller et al., 2019). ELOVL4 mutations cause autosomal dominant spinocerebellar ataxia (type 34; SCA34), erythrokeratodermia variabilis (EKV) (Cadieux-Dion et al., 2014; Bourassa et al., 2015; Ozaki et al., 2015) and autosomal dominant Stargardt-like macular dystrophy (STGD3) (Bernstein et al., 2001; Edwards et al., 2001; Zhang et al., 2001). ELOVL7 mutations have been associated with Parkinson’s disease and multiple system atrophy (Sailer et al., 2016; Li et al., 2018; Keo et al., 2020), while ELOVL2 polymorphisms were proposed to increase the susceptibility to autism spectrum disorders (Sun et al., 2018).

Two *ELOVL5* missense mutations [(c.214C > G (p.Leu72Val) and c.689G > T (p.Gly230Val)] have been identified as the genetic cause of the Spinocerebellar Ataxia 38 (SCA38) (Di Gregorio et al., 2014). SCA38 is a rare form of autosomic dominant neurological disorder characterized by gait ataxia, nystagmus, peripheral neuropathy, hyposmia and pure cerebellar atrophy (Borroni et al., 2016). Such pathological features were recapitulated in a mouse model with a targeted deletion of the *Elovl5* gene (*Elovl5^–/–^*) showing motor coordination and balance defects, loss of the sense of smell and a selective cerebellar atrophy (Hoxha et al., 2017).

Such a variety of neurologic alterations associated with mutations of different ELOVLs, might be explained by their tissue-specific expression and tissue-specific role. While ELOVL2, ELOVL7, and ELOVL1 have a restricted CNS distribution (Tvrdik et al., 2000; Lein et al., 2007), Elovl4 expression is abundant and well characterized in both adult and perinatal mouse brain (Sherry et al., 2017). In contrast, the Elovl5 expression pattern remains largely uninvestigated so far. This work is aimed at mapping Elovl5 expression in the adult mouse CNS with the final aim to provide relevant insights to understand its role in specific CNS areas and cell types.

## Materials and Methods

### Animals

*Elovl5^–/–^* mice have been kindly provided by Dr. Moon and Dr. Horton of the UT Southwestern Medical Center (Moon et al., 2009). Brains analyzed to study the expression of Elovl5 were collected from adult (6 to 12 months old) *Elovl5^–/–^* and *Elovl5^+/–^* (C57/BL6J background) and wild type mice (C57/BL6J) of both sexes bred in our Animal Facility at NICO (Orbassano, Italy). All experimental procedures on adult mice and on pups have been approved by the Ethical Committee of the University of Torino and authorized by the Italian Ministry of Health (authorization number: 161/2016-PR and 510/2020-PR).

### Tissue Preparation

To assess Elovl5 expression in brain slices mice were anesthetized using a cocktail of ketamine (100 mg/kg body weight) and xylazine (10 mg/kg body weight) via intraperitoneal injection. The mice were intracardially perfused initially with a physiological solution (NaCl 0.9%) and then with 4% paraformaldehyde in 0.12 M phosphate buffer, pH = 7.2–7.4. Following perfusion, the brain and the spinal cord were removed and stored at 4°C for 24 h immersed in the same fixative and later transferred to a solution made of 30% sucrose in 0.12 M phosphate buffer for few days. Perfused brains and spinal cords were embedded in paraffin, cut into 5 μm-thick sagittal slices and mounted on superfrost glass slides (Fisher Scientific, Göteborg, Sweden) with Tris-glycerol supplemented with 10% Mowiol (Calbiochem, LaJolla, CA, United States).

To assess Elovl5 expression in glial cells *in vitro*, postnatal day 2 (P2) pups were cryoanesthetized in melting ice. The experimental plan was designed according to the guidelines of the NIH, the European Communities Council (2010/63/EU) and the Italian Law for Care and Use of Experimental Animals (DL26/2014). The study was conducted according to the ARRIVE guidelines.

### Magnetic-Activated Cell Sorting (MACS) Isolation of Oligodendrocytes and Cell Culture Procedures

To assess *Elovl5* expression in glial cells *in vitro*, oligodendrocyte progenitor cells (OPCs) were isolated from P2 mouse brain (C57/BL6J background) by Magnetic-Activated Cell Sorting (MACS; Miltenyi Biotech GmbH, Bergisch Gladbach, DE) as in Boda et al. (2015). After tissue dissociation with the Neural Tissue Dissociation Kit P (Miltenyi Biotech GmbH, Bergisch Gladbach, DE), mouse OPCs were enriched by positive selection using an anti-PDGFRα antibody conjugated to magnetic beads, according to the manufacturer’s instructions (Miltenyi Biotech GmbH). MAC-sorted OPCs were then plated onto poly-D-lysine (1 μg/ml, Sigma-Aldrich, Saint Louis, MS, United States) coated glass coverslips in a proliferative medium including Neurobasal, 1X B27 (Invitrogen, Milan, Italy), 2 mM L-glutamine (Sigma-Aldrich, Saint Louis, MS, United States), 10 ng/ml PDGF-BB, and 10 ng/ml human bFGF (Miltenyi Biotech GmbH, Bergisch Gladbach, DE), or in differentiative medium including Neurobasal, 1X B27 (Invitrogen, Milan, Italy), 2 mM L-glutamine (Sigma-Aldrich, Saint Louis, MS, United States), 0.5 nM triiodothyronine (T3; Sigma-Aldrich). In all cases, purity of the MACS-selected OPCs was verified by immunocytochemistry (more than 95% of the cells were NG2^+^ at 6 h post-plating). Microglial cells have been obtained by orbital shaking method from mixed glial cell cultures and polarized toward either a proinflammatory (M1) or pro-regenerative (M2) phenotype by incubating with TNF-α (20 ng/ml) or IL-4 (20 ng/ml) (R&D, Milan, Italy) for 48 h, as described in Lombardi et al. (2019).

### Quantitative RT–PCR

Total RNA from cell cultures was extracted with the Directzol RNA Miniprep kit (Zymo Research, Irvine, United States), and reverse transcribed to cDNA with the High-Capacity cDNA Archive kit (Thermo Fisher Scientific, Waltham, United States). Quantitative Real Time RT-PCR was performed as described in Sacco et al. (2010), with the pre-developed Taqman assay #Rn01446631_m1 (Thermo Fisher Scientific, Waltham, United States). A relative quantification approach was used, according to the 2-ddCT method. β-actin (Taqman assay #Rn00667869_m1; Thermo Fisher Scientific, Waltham, United States) was used to normalize expression levels.

### XGal Staining

To assess *Elovl5* expression we first mapped β-galactosidase activity in homozygous and heterozygous *Elovl5* knock-out mouse line where lacZ was included as a reporter under the control of Elovl5 promoter. Floating slices (30 μm-thick brain sagittal slices) were incubated overnight at 37°C with a solution containing 1 mg/ml X-gal, 0.02% Nonidet P-40, 0.01% sodium deoxycholate, 2 mM MgCl2, 5 mM potassium ferricyanide, and 5 mM potassium ferrocyanide (pH = 7.5). Subsequently, the sections were mounted on gelatin-coated slides and let air dry overnight. The next day the sections were counter-stained with Nuclear Fast Red: mounted series were washed for 2 min in distilled water and then stained in 0.1% NFR (nuclear fast red) solution for 2 min. Sections were then rinsed again in distilled water for 2 min, and subsequently dehydrated using a series of alcohols: 50% (2 min), 75% (2 min), 90% (2 min), and 100% (2 min). Afterward, the gelatin- coated slides were immersed in xylene for 5 min and finally a clear glass coverslip was applied using a permanent mounting medium.

### Immunofluorescence Reactions

Slides were incubated at 60°C for 30 min to allow the embedded tissue to firmly adhere to the glass slide. Following, paraffin was removed by immerging three time the glass slides in xylol (3 min each) and the tissue was hydrated using a series of alcohols with decreasing graduation: 99% (three washes of 5 min each), 95% (three washes of 1 min each) 70% (three washes of 1 min each). Slides were washes twice in H_2_O (1 min each) and placed for 40 min at 95°C in citrate buffer (pH = 6.0) for antigen retrieval. Following the antigen retrieval, sections were washed twice in PBS (two quick washes) and then incubated with blocking buffer (Normal Goat Serum 5%, PBS 0.12 M pH = 7.4 and 1% Triton X100). Following, slides were washed with PBS for three times and then incubated with a cocktail containing: monoclonal primary anti-Elovl5 antibody made in rabbit (1:300 NBP2-33500, Novus biologicals, Centennial, United States), 1.5% NGS and PBS+ overnight at room temperature (RT) in a humid dark chamber. The following morning the tissues was washed three times with PBS (5 min each) and incubated with biotinylated secondary antibody anti rabbit (1:200, Vector Labs, Burlingame, United States) for 2 h. Slides were then washed three times with PBS (5 min each) and incubated with streptavidin-HRP (1:100, PerkinElmer, Milano, IT), and PBS+ for 1 h at RT. Following the incubation, sections were washed three time in PBS (5 min each) and incubated with Tyramide-FITC (1:100 PerkinElmer, Milano, IT) for 5 min at RT in the humid dark chamber. After a 5-min incubation with DAPI (1:1,000, Fluka, Saint Louis, United States), when dry, glass coverslip was applied using Mowiol (Calbiochem, LaJolla, CA, United States).

For immunocytochemistry on cell cultures, after 7 days *in vitro* cells were fixed for 20 min at RT with 4% PFA in 0.1 M PB and labeled with anti-AN2 (rat homologue of NG2, 1:100; kind gift of Miltenyi Biotech GmbH, Bergisch Gladbach, DE, and Prof. J. Trotter, Johannes Gutemberg University of Mainz, DE), -MBP (Smi-99 clone, 1:1,000 Sternberger), -Iba1 (1:1,000; Wako Chemicals, Richmond, VA), -Elovl5 (1:300; NBP2-33500, Novus biologicals, Centennial, United States) antibodies overnight at 4°C in PBS with 0.25% Triton-X. Then, coverslips were incubated with Alexa488- and Alexa555- conjugated secondary antibody (Molecular Probes, Eugene, Oregon) for 1-h RT. After a 5-min incubation with DAPI (1:1,000, Fluka, Saint Louis, United States), coverslips were mounted with Tris-glycerol supplemented with 10% Mowiol (Calbiochem, LaJolla, CA, United States).

### Images Acquisition and Processing

Slides were scanned with Slide-Scanner Axiscan Z1 (ZEISS, Oberkochen, DE) both at low and high magnification (5x and 20x). Images obtained were then modified and adapted in color, contrast and brightness with Zen Software 2.1 (ZEISS, Oberkochen, DE). All the structures were analyzed and recognized by using the Interactive Atlas Viewer [IAV; Allen Human Brain Atlas–Allen Institute for Brain Science. Mouse Brain Atlas (internet)^1^.].

Whereas, cell coverslipped cultures were examined using an E-800 Nikon microscope (Nikon, Melville, NY) connected to a color CCD Camera and a Leica TCS SP5 (Leica Microsystems, Wetzlar, DE) confocal microscope. Adobe Photoshop 6.0 (Adobe Systems, San Jose, CA, United States) was used to assemble the final plate.

### Statistics

Statistical analyses were carried out with GraphPad Prism 7 (GraphPad sofware, Inc). The Shapiro–Wilk test was first applied to test for a normal distribution of the data. Since data were not normally distributed, Mann-Whitney *U*-test (to compare two groups) and Kruskal-Wallis test (for multiple group comparisons) were used. In all instances, *P* < 0.05 was considered as statistically significant. Histograms represent mean ± standard error (SE).

## Results

### *Elovl5* Gene Expression in the Central Nervous System

**FIGURE 1:**
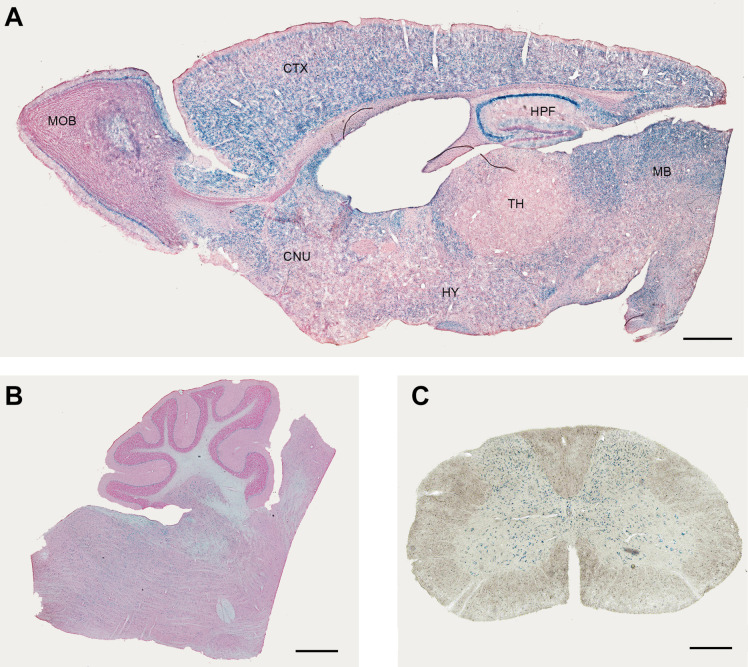
Expression of Elovl5 in the central nervous system. XGal staining (blue) indicates an ubiquitary expression of ***Elovl5*** gene in forebrain **(A)**, cerebellum and brainstem **(B),** spinal cord **(C)**. MOB, Main Olfactory Bulb; CTX, Cortex; HPF, Hippocampus; MB, midbrain; TH, thalamus; HY, Hypothalamus; CNU, cerebral nuclei. Scalebars: **(A,B)** 500 μm and **(C)** 50 μm.

In the lacZ reporter mouse, XGal staining revealed a widespread expression with some region-specific differences (Figure 1, Supplementary Figure 1, and Table 1). It is important to note that the XGal staining observed in Elovl5 lacZ reporter mouse reflected the in-situ hybridization published in Allen Mouse Brain Atlas. Specifically, in the telencephalon, a prominent labeling was found in the olfactory bulb (mitral cell layer, Figure 1A), in the neocortex, in the hippocampus (Figure 1A) and in the midbrain (Figure 1B). The thalamus showed a less pronounced, moderate XGal labeling, with the exception of a strong expression in the reticular nucleus (Figure 1A). As previously shown (Hoxha et al., 2017), in the cerebellum Purkinje cells and deep cerebellar nuclei were the most expressing cell types (Figure 1B). Interestingly, we also found several labeled cells in the cerebellar white matter, in the granular layer and in the deeper part of the molecular layer. In the spinal cord, we observed a pronounced labeling in the gray matter, in neurons of different cell body size (Figure 1C).

**TABLE 1:** Levels of expression of the ELovl5 gene (XGAL) and of the Elovl5 protein (immunofluorescence) in the central nervous system.

		XGAL	Immunofluorescence
Olfactory bulb	Glomerular layer	–	–
	Outer plexiform layer	–	SP
	Mitral cell layer	+++	+++
	Internal plexiform layer	–	–
	Granular layer	–	–
Anterior olfactory nucleus		++	++
Pyriform cortex		+++	+++
Olfactory tubercle		+	++
Islands of calleja		+++	+++
Amygdala	Cortical amygdaloid nuclei	+	+++
	Medial amigdaloid nuclei	++	++
Lateral septum		++	+
Hippocampus	Pyramidal layer	+++	++
	Stratum oriens interneurons	++	++
	Dentate gyrus	–*	–*
	Hylar neurons	++	++
	Subiculum	+	+
Neocortex	Layer I	++	++
	Layer II/III	++	++
	Layer IV	++	++
	Layer V	++	++
	Layer VI	+	+
basal ganglia	Nucleus accumbens core	+	+
	Nucleus accumbens shell	–	+
	Caudate/Putamen	++	+
	Globus pallidus	–	++
	Subthalamic nuclus	+++	++
	Substantia nigra reticulata	–	+
	Substantia nigra compacta/ventral tegmental area	+++	++
Thalamus	Reticular nuclei	++	+++
	Peripeduncular nucleus	+	++
	Subparafasciular nucleus	+	++
	Geniculate complex	+	++
	Ventral posteromedial nuclei	+	++
	Ventral posterolateral nuclei	+	++
Zona incerta		++	++
Peripeduncular nucleus		+	++
Hypothalamus	Tuberal nucleus	++	++
	Retrochiasmatic area	++	++
	Anterior hypothalamic nucleus	++	++
	Lateral hypothalamic area	++	++/+
	Supramammillary nucleus	++	++
	Mammillary nucleus	++	++
	Preoptic area	++	++
Midbrain	Inferior colliculus	+	+
	Superior colliculus	++	++
	Red nucleus	+++	+++
	Periaqueductal gray	++	++
	Midbrain reticular nucleus	++	++
Pons	Tegmental nuclei	++	++++
	Pontine gray	++	+++
	Tegmental reticular nucleus	++	+++
	Supraolivary complex	+++	+++
	Pontine reticular nuclei	++	++
Medulla	Vestibular nuclei	++++	++++
	Lateral reticular nucleus	+++	+++/++
	Medullary reticular nucleus	+++	++
	Nucleus of solitary tract	++	++
	Inferior olivary complex	++++	+++
Cerebellum	Purkinje cells	+++	+++
	Molecular layer	SP	SP
	Granular layer	SP	SP
	Deep cerebellar nuclei	++++	++++
Spinal cord	Motorneurons–laminae I to VII	+	+
	Motorneurons–lamina VIII	+++	+++
	Motorneurons–lamina X	+++	+++

Signals were rated as ++++, very high to intense; +++, intense; ++, moderate; +, low; –, not detectable; SP, labeling restricted to sparse cells. * positive cells in the subgranular region.

### Elovl5 Protein Distribution in Brain Regions

Protein localization was analyzed in wild-type mice by immunofluorescence labeling throughout different brain regions in order to define the expression pattern in diverse cell populations (Table 1). The intensity of immunoreactivity varied in different regions and neuronal populations.

#### Telencephalon

##### Olfactory Areas

**FIGURE 2:**
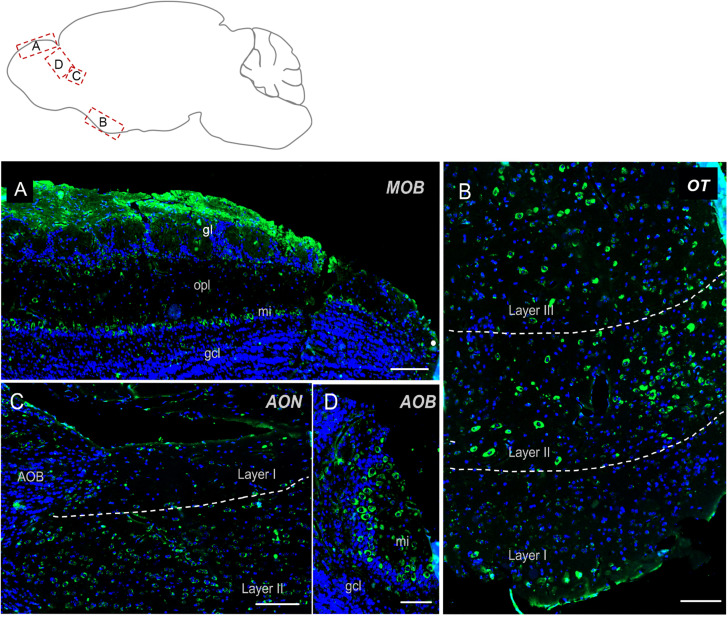
Elovl5 distribution in olfactory regions. **(A)** In the olfactory bulb, immunohistochemical labeling for Elovl5 (green) and DAPI (blue), shows a strong signal in mitral cells (mi) and in the glomerular layer (gl), while cells belonging to the granule cell layer (gcl) are negative. **(B)** The olfactory tubercle (OT) (shown at high magnification) shows a predominant expression of Elovl5 (green) in layer II and layer III neurons. **(C)** In layer II neurons of the accessory olfactory nucleus (AON) a moderate presence of the enzyme is detected. **(D)** The accessory olfactory bulb shows a similar signal as the one detected in the main bulb, with a high intensity in mitral cells (mi) and no signal in the granule cell layer (gcl). MOB, Main Olfactory Bulb; mi, mitral cells; gl, glomerular layer; opl, outer plexiform layer; gcl, granular cell layer; OT, Olfactory Tubercle; AON, Anterior Olfatory Nucleus; AOB, Accessory Olfactory Bulb. Scalebars: **(A,C)** 200 μm, **(B)** 100 μm, and **(D)** 50 μm.

The olfactory regions presented a very typical labeling (Figure 2). In the main olfactory bulb, mitral cells were found strongly positive for Elovl5 staining. In the superficial part of the outer plexiform layer small cells, likely tufted cells, showed moderate Elovl5 labeling, whereas no positive cells were observed in the granular layer (Figure 2A). The accessory olfactory bulb presented a similar expression pattern with a strong staining in the mitral cells (Figure 2D). In anterior olfactory nucleus positive cells for Elovl5 were found mainly in layer II (Figure 2C), whereas in the olfactory tuberculus in layers II and III (Figure 2D).

##### Hippocampal Formation

**FIGURE 3:**
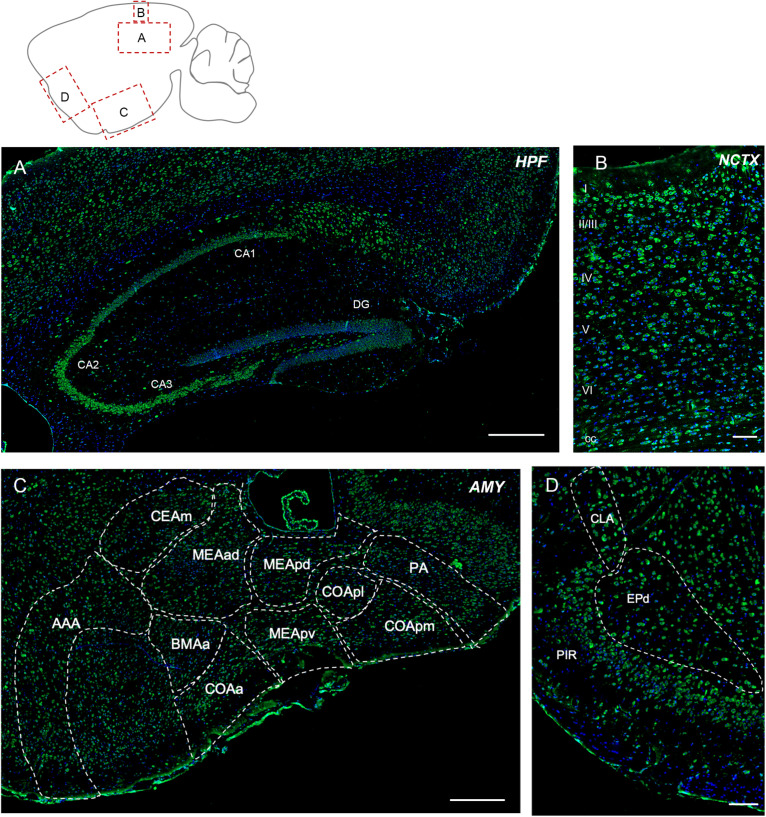
Elovl5 expression in cortical and subcortical regions. **(A)** In the hippocampus, Elovl5 (green) is strongly expressed by CA2 and CA3, in a moderate way by CA1, while it is not present in dentate gyrus. Oriens, radiatum, and hilar interneurons are strongly labeled. Cell nuclei are counterstained by DAPI (blue). **(B)** In the neocortex Elovl5 labeling is present in a moderately strong way in layers II/III, IV and V. In layer VI, neurons display a weak intensity staining. **(C)** In the amygdala Elovl5 expression is more prominent in the cortical areas than in medial ones. **(D)** The claustrum is moderately positive for Elovl5 staining, while piriform and endopiriform cortex show a strong signal. HPF, hippocampal formation; CA, Cornu Ammonis; DG, dentate gyrus; NCTX, neocortex; AMY, Amygdala; AAA, Anterior amygdalar area; CEAm, Central amygdalar area, medial part; MEAad, Medial amygdalar area, anterodorsal part; MEApd, Medial amygdalar area, posterodorsal part; MEApv, Medial amygdalar area, posteroventral part; COApl, Cortical amygdalar area, posterior part; COApm, Cortical amygdalar area, posterior part, medial zone; PA, posterior amygdalar nucleus; BMAa; Basomedial amygdalar nucleus, anterior part; PIR, piriform cortex; CLA, claustrum; EPd, endopiriform nucleus, dorsal part. Scalebars: **(A)** 500 μm, **(B,D)** 100 μm, and **(C)** 250 μm.

Elovl5 immunolabeling was expected to be extremely strong in this region, because of the high signal obtained with the XGal staining (Figure 1A). Immunofluorescence analysis confirmed a relatively strong labeling of pyramidal neurons, but with regional differences (Figure 3A). In fact, in all hippocampal fields Elovl5 labeling was clearly present in pyramidal neurons, with a gradient of intensity: the signal was stronger in CA2 and CA3 than in CA1 and subiculum, where it was just moderately intense. Several neurons of the strata oriens and radiatum were strongly labeled. Hilar neurons also showed a strong Elovl5 staining. Neurons of the dentate gyrus were negative, with the exception of a few strongly labeled cells in the subgranular zone.

##### Neocortex, Claustrum, and Amygdala

The cerebral neocortex showed a layer-specific expression pattern (Figure 3B). Cells showing moderately strong Elovl5 labeling were found in layer II/III, IV and V. In layer VI neurons displayed a weak intensity staining. In the corpus callosum, sparse positive cells were found. The piriform and endopiriform cortex showed a strong Elovl5 labeling (Figure 3C), whereas claustrum displayed a moderate intensity labeling (Figure 3C). In the amygdala, neurons showed a moderate reactivity for Elovl5 in general: more prominent in the cortical regions than in medial ones (Figure 3D).

##### Basal Ganglia

Caudate, putamen and nucleus accumbens presented a weak fluorescence signal for Elovl5 staining, with just few sparse cells with a very strong labeling. Whereas, the globus pallidus displayed a strong signal for Elovl5 immunofluorescence staining in both internal and external parts. The subthalamic nucleus was strongly labeled. The substantia nigra pars compacta and the ventral tegmental area showed a strong labeling (Table 1).

#### Diencephalon

##### Thalamus

**FIGURE 4:**
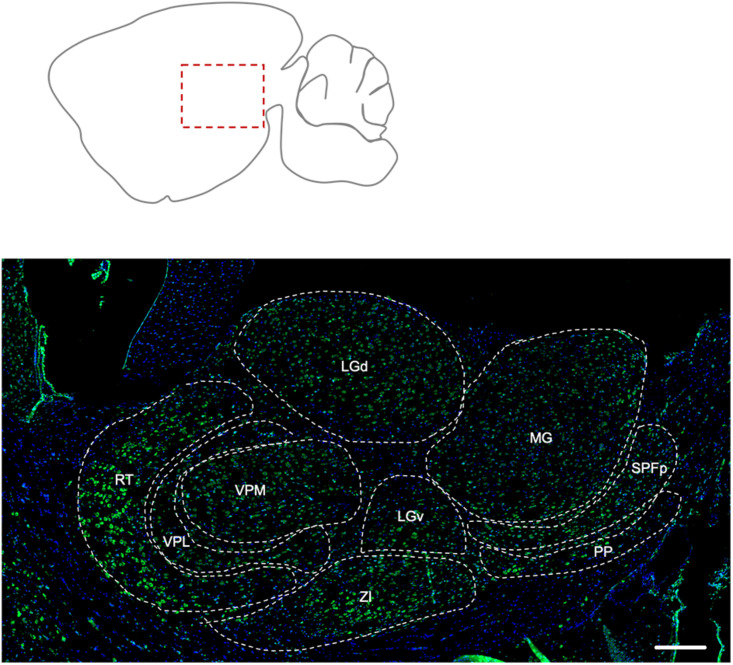
Distribution of Elovl5 in thalamus. Elovl5 (green) expression in the thalamus has a region-specific intensity. The most intense labeling is shown by the reticular nucleus. LGd, dorsal part of lateral geniculate complex; LGv, ventral part of lateral geniculate complex; MG, medial geniculate complex; SPFp, subparafasciular nucleus, parvicellular part; PP, peripeduncular nucleus; ZI, zona incerta; VPM, Ventral posteromedial nucleus of thalamus; VPL, Ventral posterolateral nucleus of thalamus; RT, Reticular nucleus of thalamus. Scale bar: 200 μm.

The reticular nucleus of the thalamus showed the most prominent labeling for Elovl5, compared to the other nuclei (Figure 4). The peripeduncular nucleus also showed a strong labeling. The parvicellular part of subparafascicular nucleus, geniculate complex and ventral posteromedial and posterolateral nuclei of the thalamus presented instead moderate immunoreactivity to Elovl5 staining (Figure 4 and Table 1). In the zona incerta there was a strong labeling (Figure 4 and Table 1).

##### Hypothalamus

In the hypothalamus, the tuberal nucleus, retrochiasmatic area and anterior hypothalamic nucleus displayed the most prominent labeling for Elovl5 compared to the other structures belonging to this region. The lateral hypothalamic area showed a heterogenous expression pattern: the immunofluorescence signal was stronger in the rostral part of the nucleus while it had a weaker intensity in the caudal part (Table 1). Supramammillary and lateral mammillary nuclei, as well as lateral preoptic area presented a moderate immunoreactivity to Elovl5 antibody.

#### Brainstem

The Elovl5 expression pattern was very complex in the brainstem. The brainstem together with the cerebellum showed the highest intensely labeled cells (Table 1).

##### Midbrain

The inferior colliculus displayed weak or no labeling for Elovl5, whereas the superior colliculus showed a moderate signal. The most prominent labeling in this region was in the red nucleus. The periaqueductal gray and the midbrain reticular nucleus displayed moderate labeling (Table 1).

##### Pons

**FIGURE 5:**
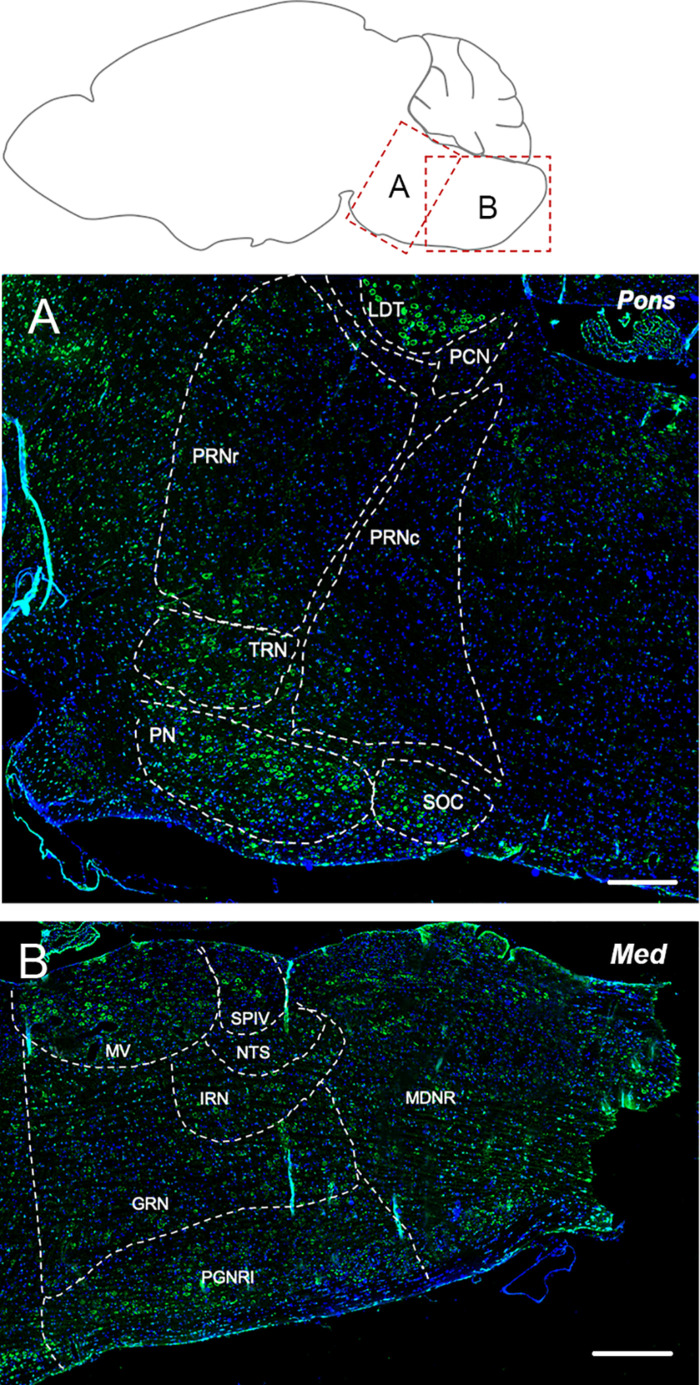
Elovl5 expression in pons and medulla. **(A)** In the pons, the most prominent labeling for Elovl5 is shown by tegmental nuclei, while pontine nuclei, tegmental reticular nucleus and superior olivary complex displayed a moderately strong signal. Medium-low intensity is shown by pontine reticular nuclei. **(B)** In the medulla, very strong labeling for Elovl5 is shown by medial vestibular nucleus and spinal vestibular nucleus. A moderate to weak signal is detected in the nucleus of the solitary tract, intermediate reticular nucleus and gigantocellular reticular nucleus. A non-homogeneous pattern of expression is present in the paragigantocellular reticular nucleus and medullary reticular nucleus. **(A)** LDT, Laterodorsal Tegmentel Nucleus; PCN, Pontine Central Nuclei; PRNc, Pontine Reticular Nucleus, caudal part; PRNr, Pontine Reticular Nucleus, rostral part; TRN, Tegmental Reticular Nucleus; PN, Pontine Nuclei; SOC, Superior olivary Complex. **(B)** MV, Medial Vestibular Nucleus; SPIV, Spinal Vestibular Nucleus; NTS, Nucleus of Solitary Tract; IRN, Intermediate reticular Nucleus; GNR, Gigantocellular Reticular nucleus; PGNR, Paragigantocellular Reticular nucleus; MDRN, Medullary Reticular nucleus. Scale bars: **(A)** 200 μm and **(B)** 250 μm.

The tegmental nuclei showed the most prominent labeling for Elovl5 (Figure 5A). Pontine nuclei, tegmental reticular nucleus and superior olivary complex displayed a strong presence of positive cells too (Figure 5A). The pontine reticular nuclei were found to be moderately and non-homogeneously positive for Elovl5 labeling (Figure 5A and Table 1).

##### Medulla

The medial vestibular nucleus and spinal vestibular nucleus presented a very strong labeling for Elovl5 (Figure 5B). The nucleus of the solitary tract, intermediate reticular nucleus and gigantocellular reticular nucleus showed moderate to weak reactivity to Elovl5 antibody. The paragigantocellular reticular nucleus and medullary reticular nucleus, instead, displayed a peculiar pattern of expression: in both areas, the Elovl5 signal was not homogeneous (Figure 5B). In the paragigantocellular reticular nucleus, the parvicellular part showed a more prominent expression compared to the magnocellular one (Figure 5B). In the medullary reticular nucleus the most intense signal was presented by neurons with bigger cell body compared to smaller ones, except for the fiber tract, in which small cells, probably oligodendrocytes, displayed an incredible high signal for Elovl5 labeling. The inferior olivary complex showed strong staining for Elovl5 (Table 1).

#### Cerebellum

**FIGURE 6:**
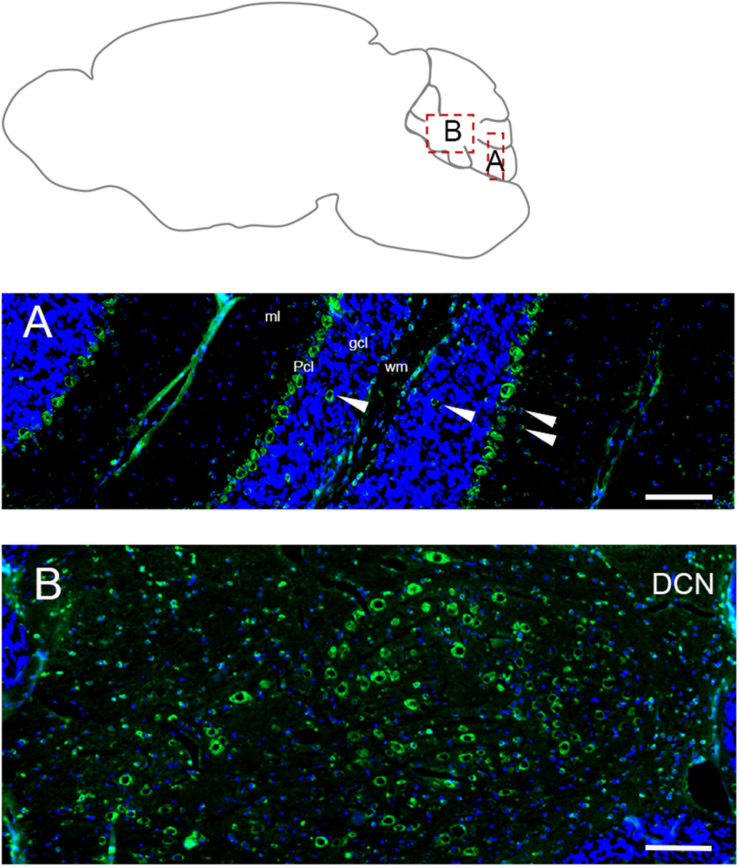
Distribution of Elovl5 in the cerebellum. **(A)** High magnification of the cerebellar cortex. Purkinje cells (Pcl) strongly express Elovl5; a moderate signal is present in sparse positive cells in white matter (wm), deeper part of the molecular layer (ml) and granular layer (gcl) (white arrow). **(B)** Prominent expression of Elovl5 (green) (DAPI in blue) by deep cerebellar nuclei (DCN). Scale bars: **(A,B)** 100 μm.

In adult cerebellum the immunofluorescence labeling for Elovl5 reflected faithfully the XGal staining (Figure 1B). Purkinje cells showed a strong positivity in cell bodies (Figure 6A) with no differences in the expression pattern between anterior and posterior lobules (Hoxha et al., 2017). The labeling was present also in some sparse neurons in the granular layer, in the deeper part of the molecular layer and cerebellar white matter (Figure 6A). Interestingly, deep cerebellar nuclei displayed the most intense labeling (Figure 6B).

#### Spinal Cord

**FIGURE 7:**
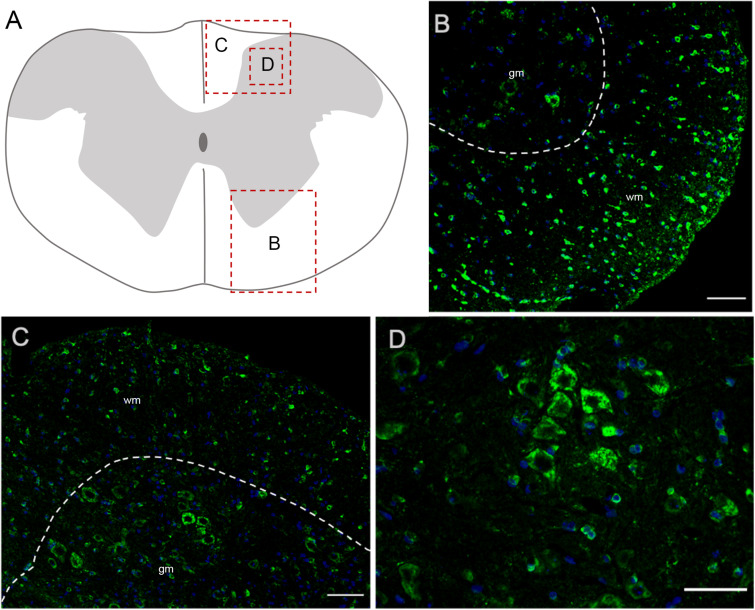
Elovl5 in the spinal cord. **(A)** Schematic representation of a spinal cord coronal section and areas magnified showed in panels **(B–D)**. **(B,C)** Elovl5 (green) is strongly expressed by small cells in the spinal cord white matter and in a moderate way by motor neurons in the gray matter. **(D)** High magnification of large neurons of the dorsal horn, positive for Elovl5 staining (green). **(B,C)** wm, white matter; gm, gray matter. Scale bars: **(B,C)** 100 μm and **(D)** 50 μm.

In the spinal cord, Elovl5 showed a well distinguishable cell-type expression pattern (Figures 7A–D). In fact, by analyzing the gray matter, motor neurons belonging to laminae VIII and IX presented a moderate intensity signal for Elovl5 staining (Figure 7B). From laminae I to VII, instead, cells displayed very weak labeling. The most prominent expression seemed to belong to glial cells in the white matter (Figures 7C,D).

### Elovl5 Expression in Glial Cells

**FIGURE 8:**
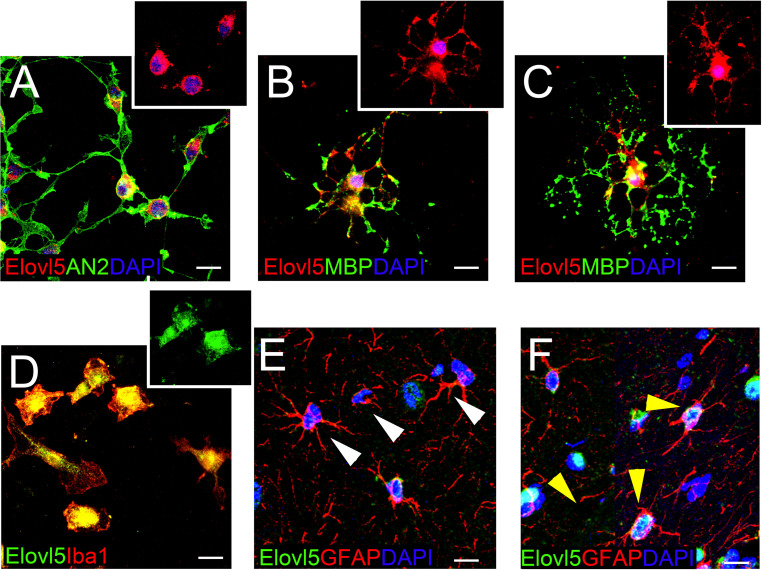
Elovl5 expression in glial cells. **(A–C)** Widespread distribution of Elovl5 (red) in cultured AN2^+^ (green) oligodendrocyte precursor cells **(A)** and in maturing **(B)** and differentiated ramified **(C)** MBP^+^ (green in B-C) oligodendrocytes. **(D)** Elovl5 (green) expression in cultured Iba1^+^ (red) microglia. **(E,F)** Sagittal brain slices showing low and heterogeneous expression of Elovl5 (green) in GFAP^+^ (red) astrocytes. White arrowheads in **(E)** point to negative cells. Yellow arrowheads in **(F)** point to Elovl5^+^ cells. Scale bars: 10 μm.

**FIGURE 9:**
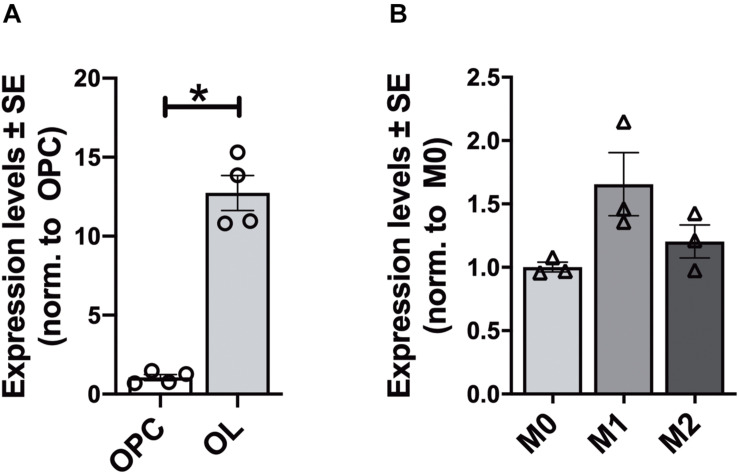
Gene expression analysis of Elovl5 in glial cells. **(A)** Elovl5 expression in oligodendrocytes (OL, *n* = 4) compared to immature oligodendrocyte precursor cells (OPC, *n* = 4) (*P* < 0.05 Mann-Whitney *U*-test). **(B)** Elovl5 levels in microglial cells in resting (M0, *n* = 3), proinflammatory M1 phenotype (*n* = 3) and proregenerative M2 phenotype (*n* = 3). The expression levels were compared to the microglial cells in resting state. *P < 0.05.

In each analyzed region of the CNS Elovl5 immunoreactivity was not restricted to neurons, but rather comprises glial cells, whose immunophenotyping was in most cases hampered by the slice treatments required for anti-Elovl5 immunostaining (see section “Materials and Methods”). Thus, in order to identify Elovl5 positive glial cell types, double immunofluorescence staining was performed on primary cell cultures and on sagittal mouse brain sections. Elovl5 was expressed by virtually all immature oligodendrocyte precursor cells (identified by the expression of the chondroitin sulfate proteoglycan AN2; Boda et al., 2011) and by oligodendrocytes (expressing the myelin basic protein, MBP) *in vitro* (Figures 8A–C). Notably, Elovl5 mRNA level appeared remarkably increased in oligodendrocytes compared to immature OPCs (Figure 9A, *P* < 0.05 Mann-Whitney *U*-test), suggesting Elovl5 participation in oligodendroglia maturation. Elovl5 immunolabeling was also found in microglial cells, identified by expression of ionized calcium-binding adapter molecule 1 (Iba1) (Figure 8D). In these cells, Elovl5 mRNA expression showed a non-significant tendency to increase when microglia were polarized toward a proinflammatory M1 phenotype, compared to resting microglia (Figure 9B). In contrast, microglia polarization toward a proregenerative M2 phenotype was not associated to changes in Elovl5 transcript levels (Figure 9B). Finally, while most astrocytes showed no or little immunolabeling for Elovl5, a subpopulation of glial fibrillary acidic protein (GFAP) positive astrocytes was found positively marked (Figures 8E,F).

## Discussion

The present study provides for the first time, to our knowledge, a comprehensive description of Elovl5 cell- and region-specific localization in the adult mouse brain. By immunohistochemistry, we showed that there are no rostro-caudal and ventro-dorsal differences in the expression of Elovl5 throughout the brain. Interestingly, big soma projecting neurons (e.g., mitral cells, cerebellar Purkinje cells, and cortical/hippocampal pyramidal cells) and glial cells showed a prominent expression of Elovl5.

XGal staining and immunohistochemistry revealed subtle discrepancies between Elovl5 promoter activity and Elovl5 protein expression. Indeed, we observed that the XGal staining was very strong and homogeneous in the hippocampal pyramidal neurons, while the fluorescent signal was only moderately intense in CA2 and CA3 pyramidal cells and lower in the other hippocampal fields. On the other hand, in the thalamus, XGal analysis showed a low signal, while the fluorescent signal was stronger in all thalamic nuclei. These results suggest the existence of transcriptional or post-transcriptional regulation of Elovl5 expression. Notably, it is reported that estrogens can act as negative regulators of miRNAs which downregulate Elovl5 at a post-trascriptional level (Zhang et al., 2017).

Elovl5 distribution in distinct CNS areas and in specific cell types suggests a unique role of this enzyme in the local synthesis of PUFAs subserving specific neuronal/glial processes and CNS functions. In the olfactory areas of the telencephalon, mitral cells showed the most prominent expression of Elovl5 protein, in contrast with a lack of labeling in the granular layer. Furthermore, strong positivity for Elovl5 was found in pyriform cortex, olfactory tubercle and anterior olfactory nucleus, which are part of the olfactory system. These findings are in line with the loss of the sense of smell found in SCA38 patients and in *Elovl5^–/–^* mice (Borroni et al., 2016; Hoxha et al., 2017), suggesting a crucial role of Elovl5 in olfaction, in line with the reported importance of PUFAs availability for a correct olfactory discrimination and a proper olfactory tissue integrity (Greiner et al., 2001; Le Bon et al., 2018; Khoury et al., 2020).

A pattern similar to the olfactory bulb was also observed in the cerebellar cortex. Purkinje cells were strongly positive for Elovl5, whereas granular cells were negative. This expression pattern well correlates with the gait abnormality and balance deficits observed in SCA38 patients with *ELOVL5* mutation and in the *Elovl5^–/–^* mouse model (Borroni et al., 2016; Hoxha et al., 2017). By studying this pathological mouse model, it has been shown that the absence of *Elovl5* in Purkinje cells led to a shrinkage of the dendritic tree causing molecular layer atrophy (Hoxha et al., 2017). This evidence suggests an important role of Elovl5 for the correct maintenance of Purkinje cell dendritic morphology, a necessary requirement for cerebellar function.

In the hippocampus, Elovl5 showed a peculiar expression pattern: labeling was specific for pyramidal cells and interneurons of the strata oriens and radiatum. The involvement of PUFAs in hippocampal synaptic plasticity has been largely described in the literature (Fukaya et al., 2007; Cutuli et al., 2014; Thomazeau et al., 2017) suggesting that the Elovl5 expression in this region is required for proper functioning and refinement of the circuits.

The characterization of Elovl5 expression in the brainstem was challenging because of the huge density of cells and nuclei. In general, the most prominent labeling was shown by nuclei functionally and anatomically connected to the cerebellum (e.g., the red nucleus in the midbrain and the vestibular nuclei in the medulla).

An interesting finding is that Elovl5 expression is not restricted to neurons but is extended to glial cells. The expression by both mature and immature oligodendrocytes suggests that Elovl5 downstream products might be important players in myelination and other oligodendroglia functions.

Along with oligodendrocytes, microglial cells showed a prominent expression of Elovl5, consistent with the observation that some Elovl5 downstream products, omega-3 PUFAs, play a major role in the resolution of neuroinflammation and in enhancing beneficial immune response (Ebert et al., 2009; Chen et al., 2014; Joffre et al., 2019). Astrocytes instead showed no or little Elovl5 immunolabeling. However, a subpopulation of astrocytes was found positively marked, suggesting a local regulation of fatty acid production that could be possibly exploited for signaling or expansion of local membranes in astrocytes (Sakers et al., 2017). Furthermore, it has been recently shown that, while undergoing a neurogenic program, some astrocytes need to upregulate genes encoding for enzymes essential for lipid metabolisms, including Elovl5 (Magnusson et al., 2020).

The expression pattern of Elovl5 in the CNS is similar to the expression of Elovl4 (Sherry et al., 2017). Mutations of ELOVL5 or ELOVL4 cause spinocerebellar ataxia 38 and 34, respectively (Ozaki et al., 2015; Borroni et al., 2016) with common neurological symptoms which might be explained by the regional expression similarity between the two enzymes. Interestingly, both enzymes are expressed in neurons and glial cells. Elovl5 is expressed by oligodendroglial cells, microglia and is heterogeneously *in vivo* expressed by astrocytes while Elovl4 is reported to be weakly expressed by astrocytes and presumably by oligodendrocytes (Sherry et al., 2017).

However, their cell-specific expression differs in some brain areas. In the cerebellum Elovl5 is expressed by Purkinje cells but not granule cells while Elovl4 is expressed mainly by granule cells with a low positivity in Purkinje cells (Sherry et al., 2017). Furthermore, in the olfactory bulb Elovl5 is expressed by mitral cells but not by granule cells while Elovl4 is expressed the most by granule cells with a low expression by mitral cells (Sherry et al., 2017). It is not clear how this difference in expression determines the onset of some neurological symptoms which are specific for SCA38 or SCA34, for instance the hyposmia in the SCA38 patients (Borroni et al., 2016).

Overall, the region-/cell-specific expression of Elovl5 suggests a special requirement of local production of VLCF with signaling or structural functions, in specific neuronal and glial cell types. However, further functional studies are needed to dissect the context-dependent role of Elovl5 and of its downstream products in neural cell physiology.

## Data Availability Statement

The original contributions presented in the study are included in the article/Supplementary Material, further inquiries can be directed to the corresponding author/s.

## Ethics Statement

All experimental procedures on adult mice and on pups have been approved by the Ethical Committee of the University of Torino and authorized by the Italian Ministry of Health (authorization numbers: 161/2016-PR and 510/2020-PR).

## Author Contributions

IB, FT, and EH supervised the experiments, data analysis, wrote the manuscript, and contributed to the conception of the work and to the discussion of the results. IB, FM, EB, and EH performed the experiments and data analysis. All authors approved the final version of the manuscript.

## Conflict of Interest

The authors declare that the research was conducted in the absence of any commercial or financial relationships that could be construed as a potential conflict of interest.

## Footnotes

1 http://mouse.brain-map.org
