# Supplementary material for: Elovl5 Expression in the Central Nervous System of the Adult Mouse
Source: Front Neuroanat. 2021 Apr 29;15:669073. doi: 10.3389/fnana.2021.669073 (PMC8116736; doi:10.3389/fnana.2021.669073)

**Supplementary Figure 1. Expression of *Elovl5* in central nervous system of heterozygous mice. (A)** XGal staining (blue) indicates a widespread expression of *Elovl5* gene in heterozygous mice encephalon. **(B)** Higher magnification of main olfactory bulb (MOB) showing Elovl5 expression in mitral cells (mi), while granule cells layer (gcl) and outer plexiform layer (opl) show no signal. **(C)** In the hippocampus (HPF) Elovl5 expression is shown by CA1 (Cornu Ammonis 1) and weakly by CA2 and CA3. Dentate gyrus (DG) is negative for XGal staining. **(D)** Thalamus (TH) shows very low signals for Elovl5 labelling. **(E)** Higher magnification of neocortex (NCTX). Elovl5 is expressed in a moderate way in layers II/III, IV and V. In layer VI, neurons display very weak intensity staining. **(F)** In the cerebellum (CRB) Elovl5 is expressed in Purkinje cell layer (Pcl), while granule cell layer (gcl), molecular layer (ml) and white matter (wm) show no detectable signal for XGal staining. **(G)** Deep cerebellar nuclei (DCN) strongly express Elovl5. **(B)** MOB, Main Olfactory Bulb; mi, mitral cells; opl, outer plexiform layer; gcl, granular cell layer; **(C)** HPF, hippocampal formation; CA, Cornu Ammonis; DG, dentate gyrus; **(D)** NCTX, neocortex; **(E)** and **(F)** CRB, cerebellum; ml, molecular layer; Pcl, Purkinje cell layer; gcl, granule cell layer; wm, white matter; DCN, deep cerebellar nuclei. Scalebars: **(A)**, **(C)** and **(D)** 500 µm, **(B)** 50 µm, **(E)** 200 µm, **(F)** and **(G)** 100 µm.


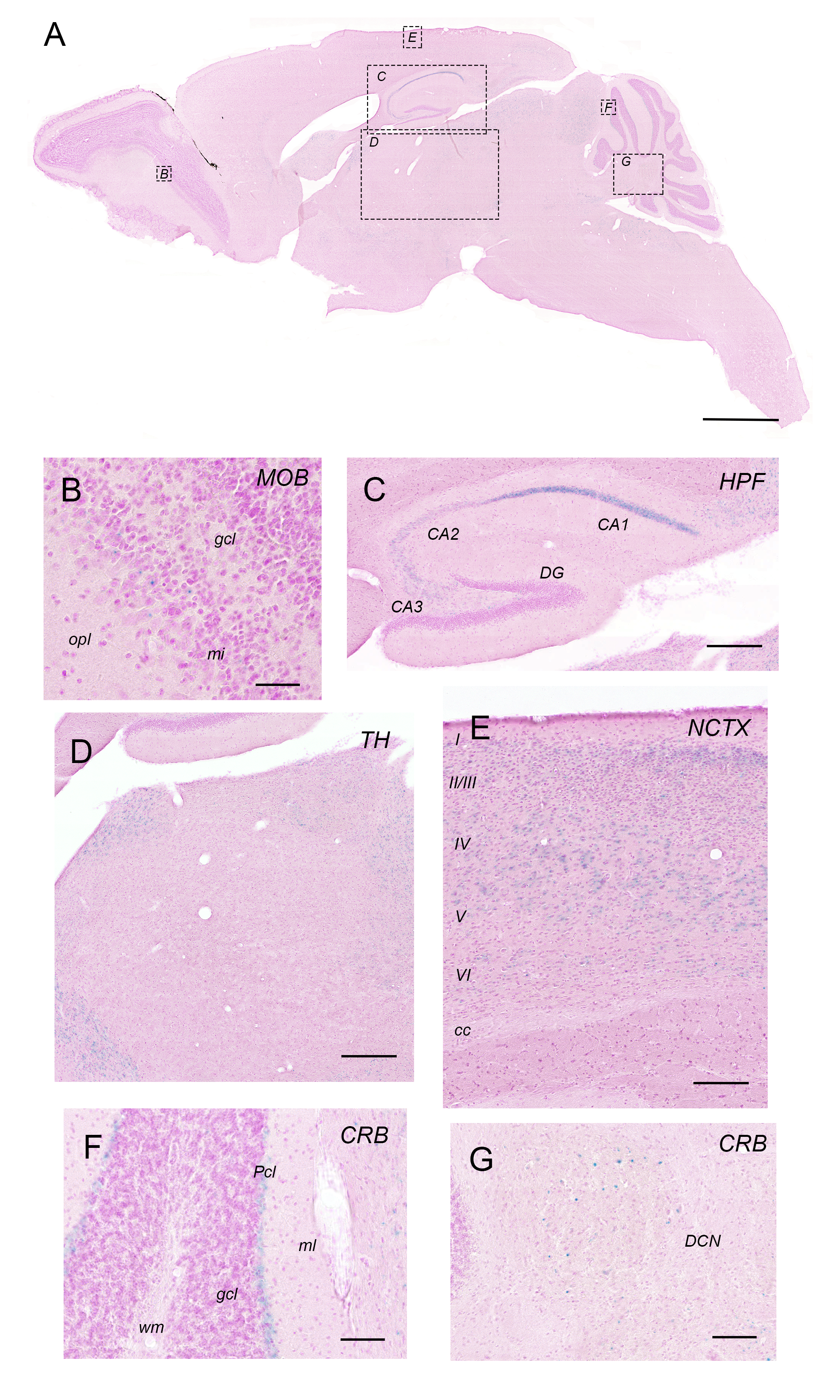

Supplement: Supplementary file 1 [file Data_Sheet_1.docx]
